# Automated solid-liquid extraction system

**DOI:** 10.1155/S1463924678000085

**Published:** 1978

**Authors:** H. Bartels, R. D. Werder, W. Schürmann, R. W. Arndt

**Affiliations:** 1 Ciba Geigy Corporation Basle CH4000 Switzerland; 2 Mettler Instrumente A G Greifensee CH8606 Switzerland


### Automated solid-liquid extraction system

**H. Bartels and R. D. Werder**
*Ciba Geigy Corporation, CH4000, Basle, Switzerland*

**W. Schürmann and R. W. Arndt**
*Mettler Instrumente AG, CH8606 Greifensee, Switzerland.*

**Automation** of wet chemical analytical procedures, particularly titration, photometry and chromatography, have wide application in industrial laboratories. Very often some sample pretreatment must be carried out to transfer the sample into a suitable state for the measurement or chromatographic process. In industrial analysis, samples are very often solids which must be reduced in size and dissolved in a suitable solvent. In manual analysis the size reduction is effected by grinding either using a pestle and mortar or a suitable mill, the crushed sample is then dissolved and excess residue filtered off. Instruments which automate these processes to a greater or lesser extent have been designed, in each the solvent is added so that both grinding and dissolution are carried out simultaneously. Of these neither the Assayomat [1] nor the Beckmann AMA 40 [2] has been successfully used and the solid prep [3] is limited to use with large batches of similar samples in conjunction with AutoAnalyzer systems [4].

With the increasing use of automated measurement in wet chemical analyses the associated manual sample preparation then becomes the most significant time consuming tedious and least satisfactory operation. It is, therefore, highly desirable to design and develop a highly reliable universal solid liquid extraction device. To be practically useful the device should encompass a number of other features: It should be able to handle a wide range of solid samples and extract them with the wide variety of solvents normally used in manual analysis. It should mimic manual processes so as not to compromise the analyst into modifying his techniques. Large batches of similar samples or more varied batches should be incorporated with equal dexterity. The device should be suitable to operate both in a stand-alone configuration and to be integrated into a more complex automatic system i.e. coupled to solution handling, spectro-photometers or electranalytical techniques [5].

Provided that a high degree of reliability is designed into the device it should improve the analytical reproducibility, any cross contamination between samples should be minimised. Good reproducibility can be obtained by reducing the sample size to the order of a few um in a short time and by minimising the surface contact area, this should also be simple and easy to wash. Any cross contamination can be reduced by designing a flexible washing system which can be automatically applied. In addition the level of automation should release the highest percentage of staff time for other tasks in the laboratory.

### Principles of operation

Preliminary experimental work showed that the size reduction is as best achieved by mechanizing the classical pestle and mortar approach. Wet milling is preferable since it eliminates local overheating problems. Emptying the mortar can be easily accomplished by situating an outlet beneath the mortar and flushing solvent through it.

Solid liquid extraction can be effected by both filtration and centrifugation; both techniques have been integrated into automatic equipment [1, 2, 3, 6, 7]. Centrifugation is preferred because it is more reliable. The physical and chemical processes involved are well known and it avoids the use of consumeables. This approach has therefore been incorporated in the instrument described here.

### Control mechanism

It is essential to be able to change rapidly from one instrument setting to another. The instrument parameters are best automatically set as a series of execution parameters at the

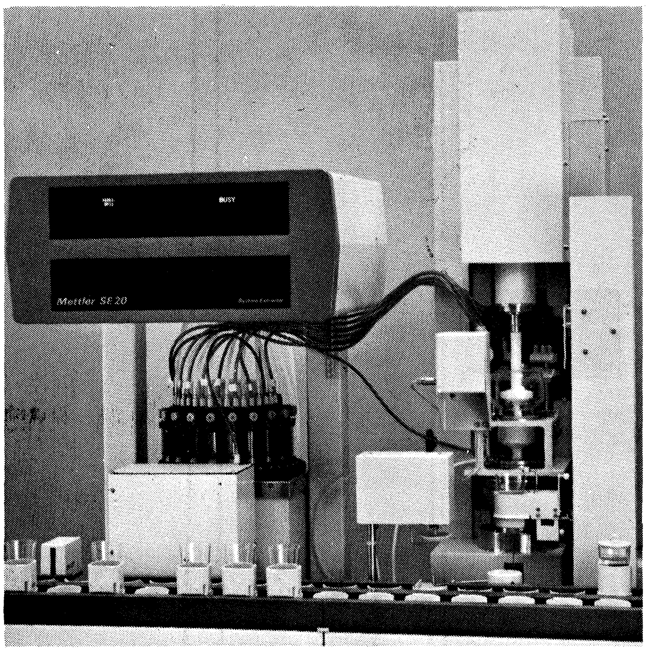

*Figure 1. View of the automated solid-liquid extractor with transport system.*

beginning of a new analytical cycle. Basically the instrument consists of two modules; the solid liquid extractor and the sample changing unit. A hierarchical control structure is preferable using two separate units to provide sequence control of each module and a third unit, either a desk calculator or a minicomputer to provide overall control and to interface to the user. A similar control system has been described in relation to an automatic titrator [8].

The solid liquid extraction unit, its control operation and maintenance are described in the foregoing sections. In combination with automatic dilutors, automatic liquid extraction devices and various measurement techniques completely automated analytical systems can be configured. Results are also presented using the device in a number of varied applications.

### Instrumentation

The solid liquid extractor is shown in Figure 1. The heart of the apparatus is the wet mill with an outlet for discharging crushed wet material into the continuous flow centrifuge. It can handle up to 10g of solid sample material.

The funnel shaped milling bed is constructed from highly resistant aluminium oxide. The discharge tube extends into the centrifuge. The rotor, a single piece pestle also fabricated from aluminium oxide, fits closely into the milling chamber and the lower end portum forms a seal with the milling chamber discharge pipe of the milling bed.

The pestle and its drive mechanism can be moved in a vertical plane by a separate drive mechanism. The pestle can therefore be set in four positions. These positions are illustrated in Figure 2. In position 2(i), the pestle is raised above the milling bed and samples can be introduced, the discharge tube remains closed, in position 2(ii) the sample is hammered crushed and finally ground by the action of the pestle on the milling bed. The pestle then moves to an intermediate position between these two and the sample homogenised. The pestle then moves to its highest location, the discharge tube opened and sample flushed into the centrifuge.

### Centrifuge

Like the milling bed the centrifuge, Figure 3, is also shaped in the form of a funnel. At the base of the centrifuge is a discharge tube (d) which is covered by a rotating distribution plate (a). With the distribution plate rotating at high speed suspensions fed into the centrifuge from the mill through (b) are thrown outwards towards the wall (c). Liquid creeps upwards on the centrifuge wall as shown in Figure 3, collects in the channel and is removed for subsequent analysis.

For safety purposes the centrifuge is mounted in a suitable casing. A series of nozzles (e) located at the top of the centrifuge allows it to be washed between samples. These nozzles can be connected to a number of cleaning solutions which are fed into the instrument with the distribution plate rotating at a slow speed, in this manner the clean solutions wash over the distributing plate and are removed through the discharge tube. Steam cleaning facilities are also available.

### Sample introduction

Samples in the form of pellets, flakes, granules, grains, tablets, capsules, suppositories and whole ampules can be extracted by simply weighing out an appropriate amount into a sample holder and then transferring this mechanically to the mill. Figure 4 shows a schematic arrangement of a complete system including a transport mechanism, sample cups and the mechanical hand, which transfers samples into the solid liquid extraction module. The lid of sample cup is removed, the cup lifted above the milling chamber and the sample transferred to the mill. In this position the sample cup may be rotated and excess sample washed into the mill. The mechanical hand

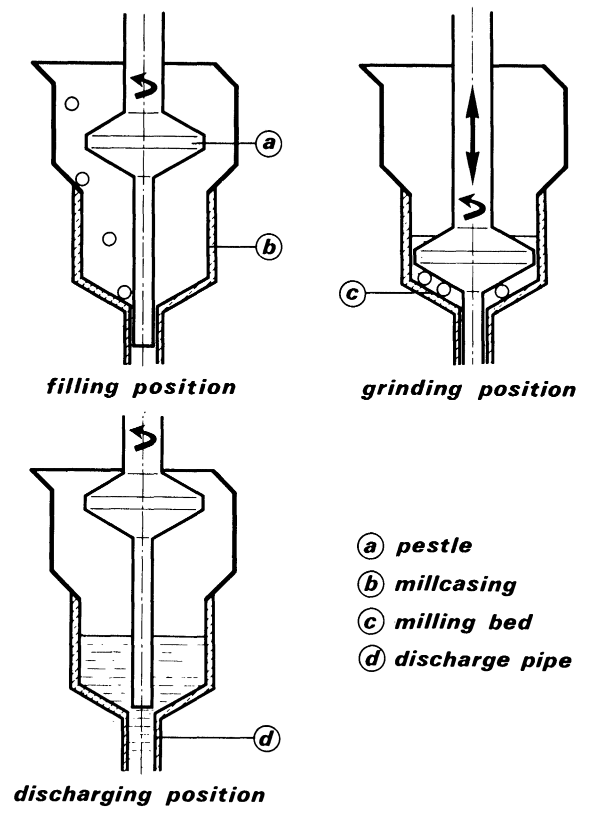

*Figure 2. Cross-sections of the mill in different operating positions.*

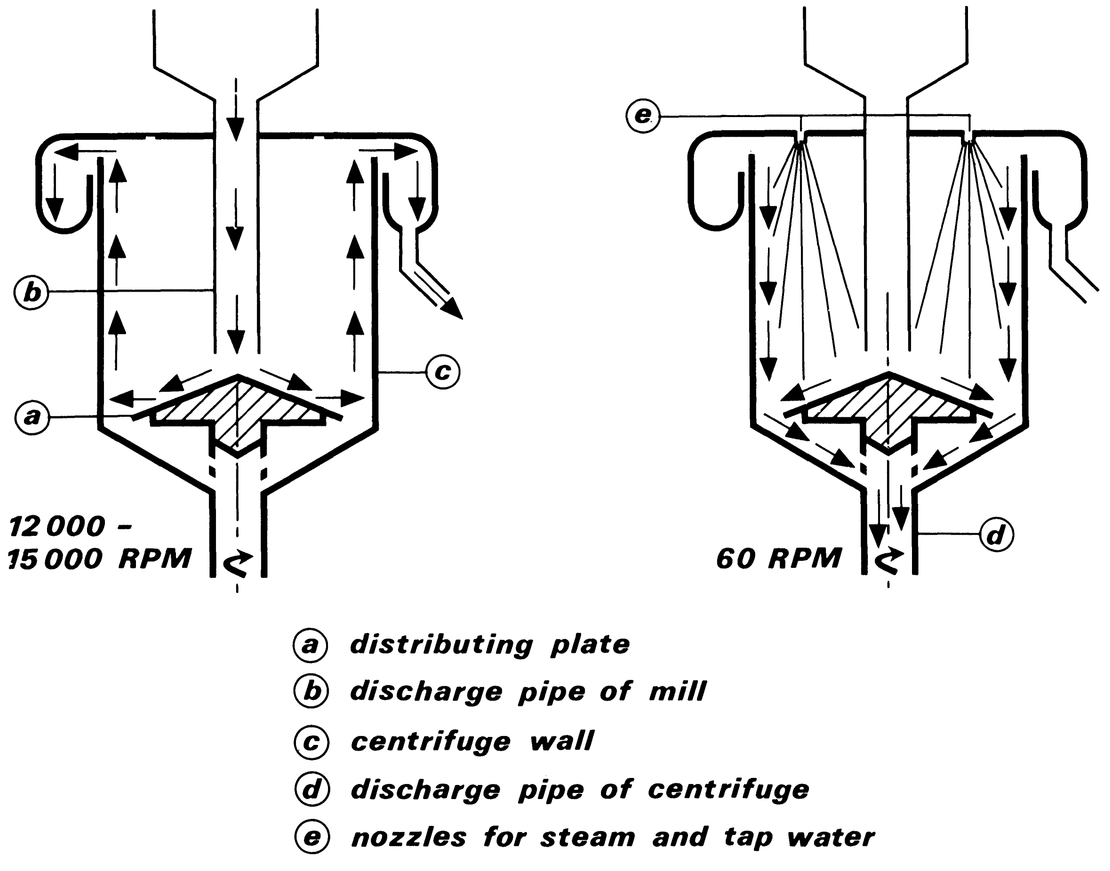

*Figure 3. Cross-section of the centrifuge in separating (left) and washing (right) cycle.*

returns the empty sample cup to the transport mechanism and the extract emerging from the centrifuge is introduced into it. Samples in the form of creams, ointments, liniments, pastes, slurries etc. can also be extracted but must first be weighed into thin walled glass tubes. These tubes filled with the appropriate amount of sample are then transferred into the mill. Solids may also be added to the sample to remove a component such as water using sodium sulphate from the system.

### Dispensing unit

Solvents and wash out solutions can be delivered from a bank of twenty burettes, capacity 20 millitres, arranged in a circle. A single motor drive can be connected to any piston via a cantilever arm, this arm and subsequently the volume of liquid delivered is adjustable using a synchronous drive motor. Three way valves at the top of the burette allows the piston to be changed from a solvent reservoir or discharged through the burette tip. Each burette tip can be used for one of the following functions: to discharge into the mill; to rinse the sample cup, or to discharge into the centrifuge for cleaning purposes.

One tip is connected to a steam generator, constructed from a hollow aluminium body heated to approximately 200°C. Steam can be discharged into the centrifuge casing to overcome difficult cleaning problems.

### Operating control

For any particular analyses the various control parameters for the extractor, milling requirements, solvents used, centrifuge conditions and the like must be entered into the control system in the form of a program of manipulations. The sequence can be given a specific program name and recalled for future operation. At the extraction stage the operator must specify whether or not the sample cup should be rinsed, with what and how much solvent. A burette can be operated either partially or repetitively. The centrifuge speed must be selected, three options are available, medium speed approximately 10,000 rpm, high speed approximately 15,000 rpm or variable speed, in this latter option the rpm desired is entered on the control panel (see Figure 5). The solvent required for milling, how much solvent, the milling time and the time allowed to transfer the suspension into the centrifuge must be entered. In addition the rinsing and washout cycle for the mill must be specified, which solvent, how much and how often.

These parameters are sufficient to control the milling and extraction process but very often a washout cycle must be specified for the centrifuge. Steam, high pressure tap water and a variety of solvents can be selected. Any residual solvent in the centrifuge can be removed by rotating the distributing plate at 15,000 rpm.

The program of operation assembled on the external control unit are transmitted to the microprocessor controls of each module and control the individual sequence of operations.

### Operation

In operation the sample is identified if necessary and then placed in the sample transport mechanism. The method of extraction to be applied is then recalled from storage using the control unit keyboard. The control unit then initiates the analytical cycle and transfers the instrument's parameters to the microprocessor control of the extractor. Figure 4 shows the hierarchical structure of the instrument design. The extractor microprocessor controls and monitors all mechanical movements and provides status signals to the control desk calculator or minicomputer.

### Configuration

Before the method can be operated with a program set up as described above the system configuration must be defined to the

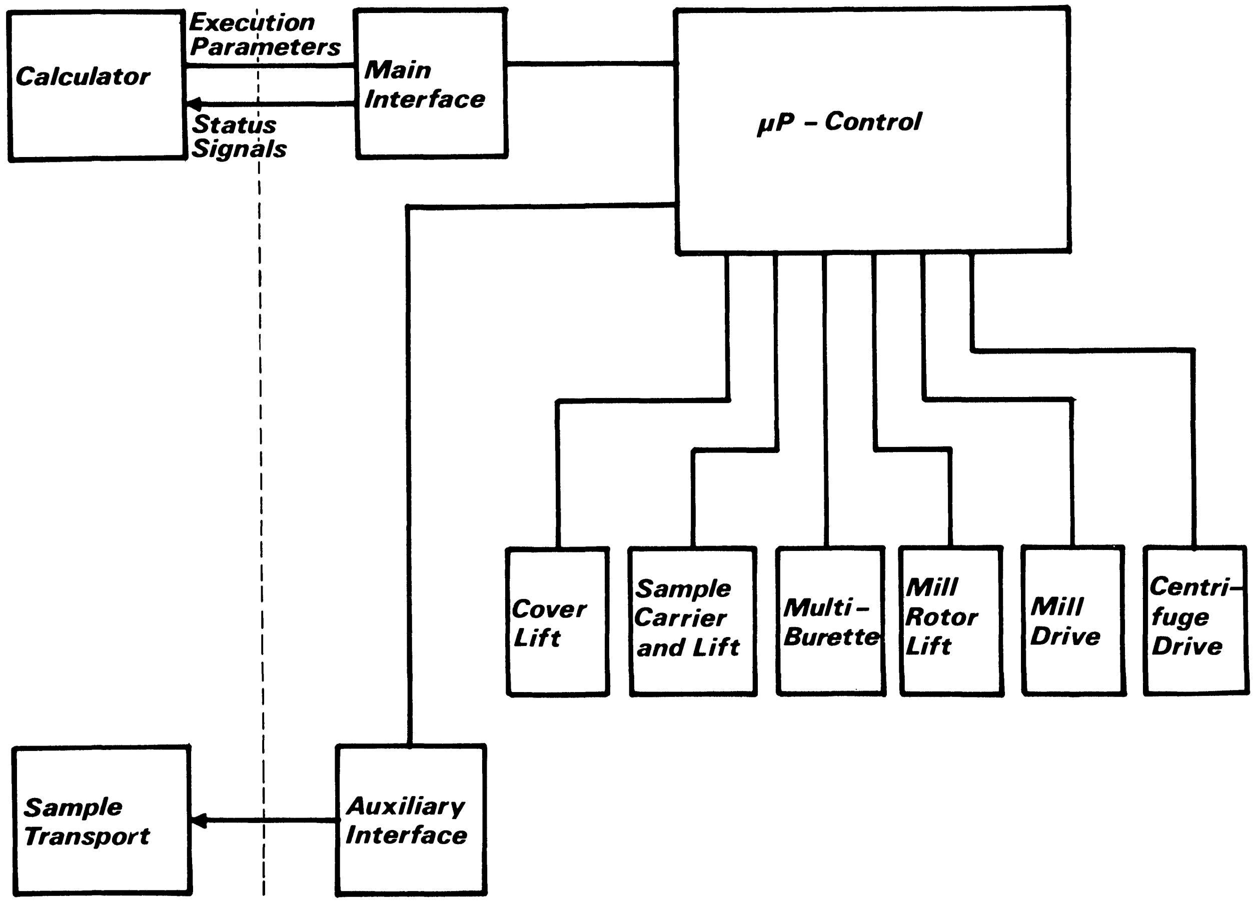

*Figure 4. Block diagram of system and extractor control.*

control system. This requires that information such as which burette contains which solvent, whether the burette is used to wash the sample cup into the mill or directly into the centrifuge etc. must be entered.

### Operating cycle

The pertinent operating parameters for the samples under test are entered, and after initialisation the sample cups are transported to the solid liquid extractor. The mill rotor is accelerated to approximately 900 rpm, moved into the appropriate position to accept the sample. The mechanical hand removes the sample cup lid, raises the cup upwards to the mill and pours the sample into the mill, rinsing if provided is also carried out at this stage. The mechanical hand then moves the sample cup to a position to receive the extract. The centrifuge is set to the appropriate operating speed. Solvents are added to the mill and the pestle lowered to hammer and grind the sample introduced into the mill bed. In this position there is only a narrow anular gap between the crushing portion of the pestle and the mill casing, solid material cannot escape from the chamber, therefore all the sample is effectively treated.

After the crushing time has elapsed the pestle is raised whilst rotation continues, further solvents may be added at this time and the stirring continued to homogenise the same (this time is variable up to 990 seconds). The pestle is then raised and the suspension fed into the centrifuge through the discharge pipe. The suspension impinges on the spinning distribution plate and is thrown outwards towards the inner wall of the centrifuge, there solid particles accummulate. The liquid extract flows upwards, is collected in the channel and fed back into its original sample cup for further processing.

To rinse the mill and elute solid particles more solvent is dispensed into the mill, the pestle is temporarily lowered into the grinding position to clean the casing mechanically. The pestle is again raised to the discharge position and the solvent flushed into the centrifuge and the solid residue washed to complete the extraction. This rinsing cycle can be repeated up to 9 times, however in practice repeating twice is adequate.

The final residue can either be washed out and discarded or re-dissolved in a suitable solvent and collected in a second sample cup in the transport mechanism and then subsequently analysed.

### Controls and maintenance

Throughout the operating cycle each operation is monitored by a microcomputer. In event of an instrument malfunction an error is displayed on the front panel, Figure 5, the specific fault light indicating the actual fault.

To carry out inspection or maintenance the instrument is switched from the 'automatic' mode into the 'maintenance' mode. In this mode a number of operations can be performed. Solvent can be dispensed from a burette which is specified with a thumb wheel switch and this must be carried out to prime the burettes whenever a solvent supply reservoir is replaced. The pestle can be raised and lowered. This facility in conjunction with the ability to latch the milling bed and its lid to the drive allows the centrifuge assembly to be inspected visually. One of two standard washout procedures can be executed, this is useful after an 'ERROR' has occurred.

The instrument controls on the front panel are only used when the instrument is in the maintenance mode. In routine use the control is via the instrument keyboard.

### Integration into an automatic system

As described above the instrument can be operated in conjunction with a sample changer and a desk calculator. It can also be integrated into an automatic system, in such two further modules are added, a diluter which manipulates the solutions and a titrator [8]. Further modules are currently being developed.

### Results

The major use of the solid extractor has been involved with the analysis of herbicides and pharmaceuticals. Most agricultural chemicals are titrated after extraction with chlorinated solvents. Depending on the concentration of the analyte in the samples, either powders or slurries, a sample size of between 0.2 to 15 g is extracted, with approximately 50 ml of solvent, ground in the mill for between 60 and 100 seconds and rinsed with solvent three times. The reproducibility of an analytical method requiring extraction and subsequent titration with perchloric acid can be judged by a standard deviation of between 0.2 to 0.5%.

The following pharmaceuticals were also extracted; tablets,

*Figure 5. Uncovered front panel with error diagnostics.*

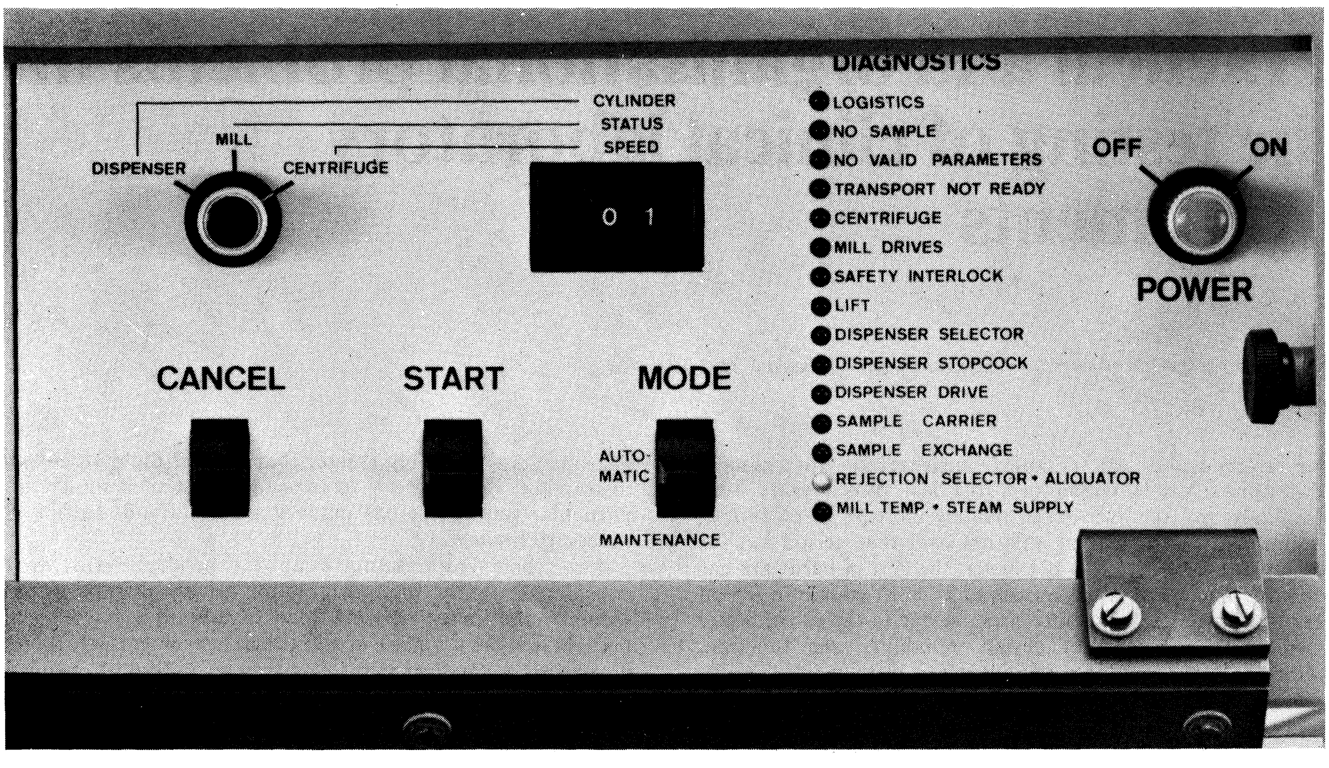

**Table 1 Some typical results obtained using the automatic solid liquid extraction module.**

| Parameter Determined | Matrix | Theoretical Value | Experimental Value | Standard Deviation | Number of samples |
|---|---|---|---|---|---|
| Titratable acid | Resin | 160mVal | 160.7mVal | 0.30% | 18 |
| Vitamin C (Ascorbic acid) | Capsule | 1000mg | 1001.2mg | 1.07% | 8 |
| Sulfonamide | Tablet | 500mg | 501.5mg | 0.41% | 6 |
| Simazine | Powder | 80% | 80.62% | 0.43% | 8 |
| Atrazine | Powder | 80% | 80.26% | 0.16% | 9 |

capsules, dry ampules, suppositories, creams, ointments and pastes using a variety of solvents such as water, aqueous dilute acids and bases, alcohol, acetone, chlorinated solvents and petroleum ether. The final measurements were carried out by direct photometry in either the UV or visible region, by indirect photometry or by a variety of titration methods, for example 0.1N solutions of HCl, NaOH, $NO_2$, $I_2$, Fe (III), Ce(IV), Ag(I), $HCO_4$, in glacial acetic acid and with TBAH. Some typical results are shown in Table 1.

Reproducibility of the automatic methods is equal or better than the equivalent manual procedure, the actual determined content of the analyte is in general equal to the manual result but in a few cases is slightly greater. A proven manual method can by simply and quickly translated into an automatic regime and results obtained within an hour. A laboratory technician can become proficient with the device with only one day's training. These features are a great advantage.

The extractor has a sample throughput of about 70 different analyses in an eight hour working day, extension into the silent hours will double this throughput. Where the analytical problem relates simply to checking sample uniformity a further doubling of sample throughput, is possible because some washing procedures can be omitted.

### Discussion

The design and construction of a highly reliable solvent extractor capable of precise analysis was made possible by close co-operation between the instrument company and a team of analysts working for a chemical manufacturer. The applicability of the device in routine analysis has been fully evaluated over an extended period of evaluation. These evaluations show that it is suitable for many applications and materials. The results obtained show that the inherent improved control over manual procedures produces increased precision of analysis.

REFERENCES

[1] C. R. Rehm, T. Urbanyi & T. J. Slone: A Versatile Automated System for the Spectrophotometric Analysis of Single Tablets. *Annals New York Acad. Sci* **153**, 640-654 (1968).

[2] D. G. Rohrbaugh & J. Ramirez-Munoz: Analytical Applications of an Automatic Material Analyzer, *Analytica Chimica Acta*, **71**, 311-320 (1974).

[3] P. Grafstein & R. Goldberg: The SOLIDPREP Sampler II and Automated Wet Chemical Analysis of Solid Samples, *Technicon International Congress 1972, Advances in Automated Analysis*, **9**, 53-59.

[4] V. Reicher: *Technicon Bibliography*, Technicon International 1973, Geneva, Switzerland.

[5] R. W. Arndt & R. Werder: Automation in Wet Chemical Analysis, Z. anal. Chemie **287**, 15-18 (1977).

[6] N. L. Alport: Automated Instruments for Clinical Chemistry, *Clin. Chem.* **15**, 1198 (1969).

[7] N. G. Anderson: Analytical Techniques for Cell Fractions, *Analytical Biochemistry* **31**, 272-278 (1969).

[8] P. V. Frueh, L. Meier, H. Rutishauser & O. Siroky: A Microcomputer-controlled Titrator for Automated Individual Analysis, *Anal. Chim. Acta* **95**, 77-106 (1977).

### Practical and organisational problems in the testing of clinical laboratory instruments

**L. B. Roberts**

*Biochemistry Department, Gartnavel General Hospital, Glasgow, G12 0YN, UK.*

**The** expenditure on complex instruments for clinical laboratories has increased over the last fifteen years both absolutely and relatively. The relative increase is, of course, conditioned by the rate of inflation over that period but for similar instruments, e.g. a pH meter, the cost of hardware has probably decreased when measured at both ends of a fifteen year time span. Absolute increases are due to the use of better instruments, in terms of design, reliability and function. Additionally the greater use of automatic instruments has also added to the absolute costs. A good example here is the move from single channel to multichannel analysers which although the capital sum of an equivalent number of single channel instruments is probably greater than that of the multichannel instrument, the necessity to expend one sum of money at a particular time point has made it more difficult to find the necessary finance.

Two other factors relating to capital expenditure must also be considered, firstly the proliferation of manufacturers and secondly the increased work load of laboratories which has necessitated the purchase of additional instruments. By present day prices a small hospital department of clinical chemistry could well have a capital investment of up to £100,000 ignoring items of equipment costing less than £100. A medium sized laboratory might have an investment up to £250,000 and a large

